# Forceps retrieval of long-dwelling permanent inferior vena cava filters: technical success and complications

**DOI:** 10.1186/s42155-026-00678-8

**Published:** 2026-06-11

**Authors:** Jacob J. Bundy, Warren A. Campbell, Matthew Abad-Santos, David Shin, Jeffrey Forris Beecham Chick, Mina S. Makary

**Affiliations:** 1 Division of Vascular and Interventional Radiology, Department of Radiology, Wake Forest Baptist Health Medical Center, Winston Salem, NC USA; 2 Division of Vascular and Interventional Radiology, Department of Radiology, University of Virginia, Charlottesville, VA USA; 3 Division of Vascular and Interventional Radiology, Department of Radiology, University of Washington, Seattle, WA USA; 4 Division of Vascular and Interventional Radiology, Department of Radiology, University of Southern California, Los Angeles, CA USA; 5 Division of Vascular and Interventional Radiology, Department of Radiology, The Ohio State University Wexner Medical Center, Columbus, OH USA

**Keywords:** Non-retrievable, Permanent, Inferior vena cava, IVC filter, Greenfield, TrapEase, Simon Nitinol, VenaTech, Bird’s Nest, Complex retrieval, Complex removal, Endovascular recovery, Endobronchial forceps, Myocardial forceps, Interventional radiology

## Abstract

**Purpose:**

To report the rate of technical success and procedural outcomes of forceps retrieval techniques for removing long-dwelling “permanent” inferior vena cava (IVC) filters.

**Materials and methods:**

Retrospective single center analysis identified 34 patients, including 19 (55.9%) males and 15 (44.1%) females, with mean age of 52 years (range 21–71 years), meeting inclusion criteria of having a “permanent” IVC filter removed. Mean filter dwell time was 112 months (range 8–337 months). Indications for “permanent” filter retrieval included iliocaval thrombosis (*n* = 23; 67.6%), strut fracture or migration (*n* = 6; 17.6%), patient request (*n* = 4; 11.8%), and future open thoracoabdominal aortic aneurysm repair (*n* = 1; 2.9%).

**Results:**

“Permanent” filters included Greenfield (*n* = 16; 47.1%), TrapEase (*n* = 10; 29.4%), Simon Nitinol (*n* = 5; 14.7%), VenaTech LP (*n* = 2; 5.9%), and Bird’s Nest (*n* = 1; 2.9%). The 3-mm endobronchial forceps were used in 30 (88.2%) patients and the 2.25-mm myocardial forceps were used in 4 (11.8%) patients. Technical success was 88.2% (30/34). Mean fluoroscopy time was 75.1 min (range 10–230 min) for the entire procedure (including venous stent reconstructions, if required). Three (8.9%) patients, with Greenfield, TrapEase, and Bird’s Nest removals, developed small IVC intimal injuries and pseudoaneurysms resulting in contained leaks with no long-term complications. One (2.9%) patient suffered a thromboembolic stroke following retrieval of a TrapEase filter which ultimately resulted in death. Mean follow-up duration was 647 days (range 27–2,265 days).

**Conclusion:**

Forceps retrieval of long-dwelling permanent IVC has a high rate of technical success with risk of injury to the IVC or thromboembolism. With appropriate discussion of the risks and benefits, a variety of long-dwelling “permanent” IVC filters can be removed.

## Introduction

Inferior vena cava (IVC) filters have become a means of reducing the risk of pulmonary embolism in patients with deep venous thrombosis with contraindication to anticoagulation or failure of prior therapy [1]. There is a steady rise in IVC filter placement with the development of retrievable filters [2]. Retrieval rates of temporary filters remain poor despite meeting criteria for removal [3]. Many clinical practices have created prospective databases to reduce follow-up filter placement to remove filters as soon as clinically indicated to reduce dwell time. As dwell time increases, filters become embedded in the IVC, resulting in failure of conventional retrieval methods and increasing complications including caval penetration, fracture, and thrombus formation [4].

Ancillary techniques which facilitate filter retrieval when conventional methods fail include balloon displacement, loop-snare entrapment, hangman techniques, dual-venous accesses, laser sheath utilization, and forceps-assisted removals [5–7]. Endobronchial forceps retrieval was demonstrated to be a valuable technique in 114 patients with a mean retrievable filter dwell-time of 1.4 years [7].

“Permanent,” or “non-retrievable,” IVC filter designs lack the apical retrieval hook and collapsible design for standard retrieval techniques, thus relegating patients to the potential associated complications of long-dwelling IVC filters. Indications for permanent filter removal include chronic pain, iliocaval thrombus, strut fracture/migration, surgical access, or resuming anticoagulation. Permanent filter retrieval techniques are not standardized and can use a combination of ancillary or forceps techniques. There are limited reports regarding the efficacy of forceps, either 3-mm endobronchial and 2.25-mm myocardial devices, for the removal of “permanent” IVC filters.

This study was conducted to determine and report the technical successes and procedural outcomes of forceps retrieval techniques, using 3-mm endobronchial and 2.25-mm myocardial devices, when utilized for the removal of long-dwelling “permanent” IVC filters.

## Materials and methods

### Patient selection

**Fig. 1:**
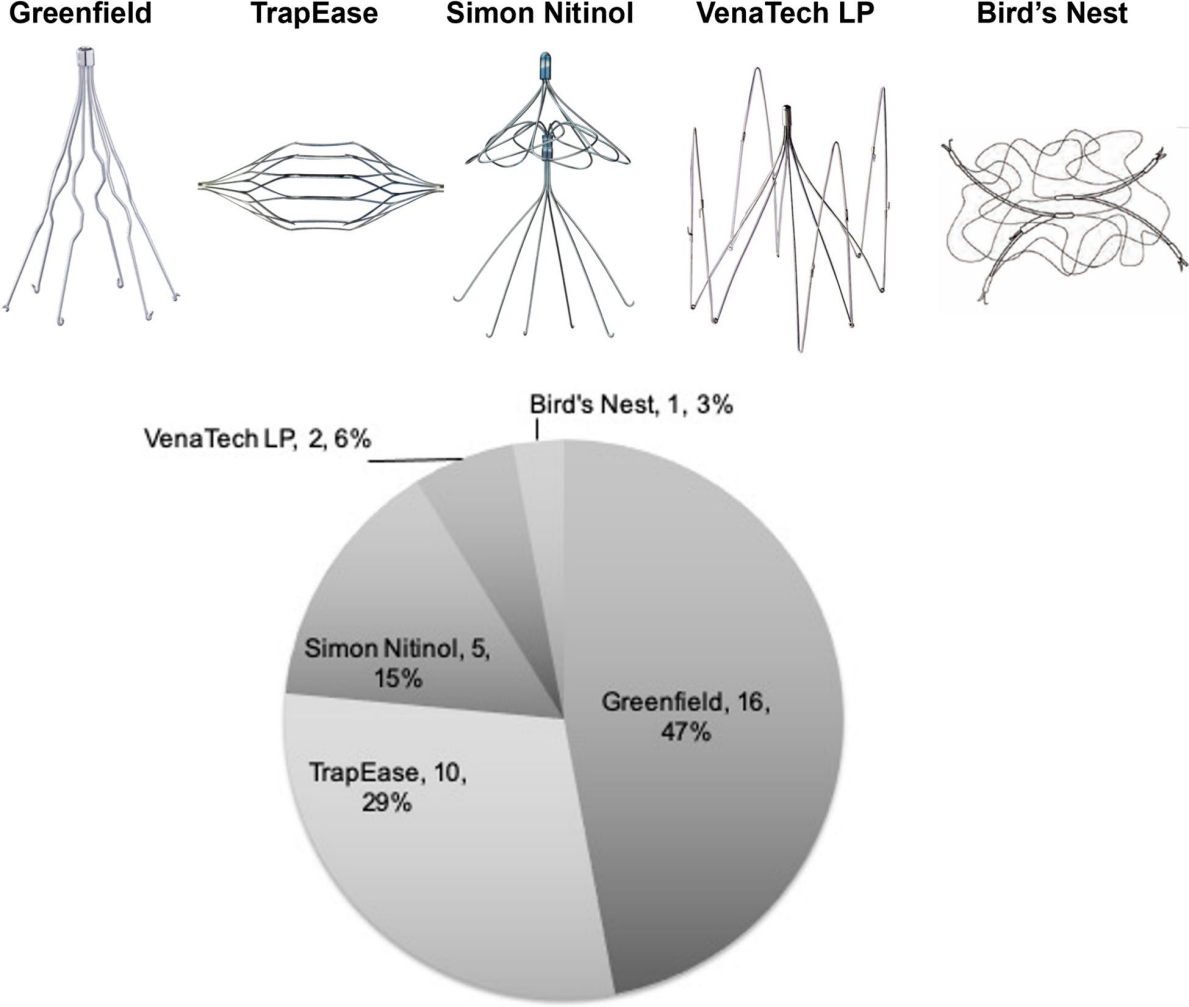
Patient selection methodology

This study was conducted with *Institutional Review Board* approval and complied with the *Health Insurance Portability and Accountability Act*. Informed consent was not required for this retrospective study. This study was assessed using the *STrengthening the Reporting of OBservational studies in Epidemiology* (STROBE) guidelines [8]. Tertiary single-center retrospective review of a prospectively maintained IVC filter retrieval database was performed [9]. This yielded a total of 284 potential patients (Fig. 1).

### Inclusion criteria

All potential retrieval patients were considered for inclusion (*n* = 284). Thirty-four of 284 (11.9%) patients underwent retrieval of a “permanent” IVC filter using either the 3-mm endobronchial or 2.25-mm myocardial forceps and were included in the final analysis.

### Exclusion criteria

Patients were excluded for the following: 129/284 (45.4%) had a retrievable (also called “temporary” or “optional”) filter which was removed, 107/284 (37.7%) had an indwelling "permanent" filter with no attempted retrieval, and 14/284 (4.9%) underwent retrieval of a "permanent" filter without the use of endobronchial or myocardial forceps.

### Patient demographics and presenting data

Patient demographics are shown in Table 1. A total of 34 patients, including 19 (55.9%) males and 15 (44.1%) females, with a mean age of 52 years (range 21–71 years) underwent retrieval of a “permanent” IVC filter with endobronchial or myocardial forceps.

**Table 1:** Patient demographics

Demographics (*n *= 34)	
Gender	*n* (%)
Male	19 (56)
Female	15 (44)
Age (years)	
Mean	52
Standard deviation	12
Range	21–71

Mean filter dwell time was 112 months (range 8–337 months). Clinical indications for filter removal are shown in Table 2. Clinical indications for “permanent” filter retrieval included symptomatic iliocaval thrombosis (*n* = 23; 67.6%), strut fracture or migration (*n* = 6; 17.6%), and planned open thoracoabdominal aortic aneurysm repair requiring venous cannulation for cardiopulmonary bypass (*n* = 1; 2.9%). In 4 (11.8%) instances, filter retrieval was performed at the patient’s request following a thorough discussion regarding the nature of the “permanent” filter and the risks involved with retrieval. Of these 4 patients, 3 were not on any form of anticoagulation and had no evidence of deep venous thrombosis. One patient had a history of bilateral pulmonary emboli and iliocaval thrombosis for which she underwent thrombolysis and was on lifelong anticoagulation.

**Table 2:** Clinical indication for “permanent” filter retrieval

Clinical Indication (*n* = 34)	*n* (%)
Iliocaval thrombosis	23 (67)
Strut fracture or strut migration	6 (18)
Patient request	4 (12)
Planned open thoracoabdominal aortic aneurysm repair	1 (3)

### Measured and defined variables

Type of “permanent” filter, forceps type (endobronchial or myocardial), sheath size (French), failed ancillary retrieval techniques employed, technical successes, fluoroscopy time (minutes), blood loss (milliliters), immediate and long-term adverse events, mortality, and follow-up duration (days) were recorded. Patients received pre-procedural CT venogram prior to retrieval. Asymptomatic patients were not routinely followed with post-procedural imaging. Anticoagulation was held per Society of Interventional Radiology (SIR) Guidelines on complex IVC filter removal.

The following “permanent” filters were included in the analysis: titanium Greenfield (Boston Scientific, Watertown, MA, USA), TrapEase (Cordis, Bridgewater, NJ, USA), Simon Nitinol (Bard Peripheral Vascular, Inc., Tempe, AZ, USA), VenaTech LP (B. Braun IS, Bethlehem, PA, USA), and Bird's Nest (Cook Medical, Bloomington, IN, USA).

Technical success of filter retrieval was defined as successful removal of the majority of the filter. Inability to retrieve all struts that fractured prior to retrieval was not considered a technical failure and considered to represent a wall-embedded chronic fragment with a very low risk of migration or embolization. All struts fractured during filter removal required extraction for technical success. When filter extraction was unsuccessful, planned future interventions were described. Complications were classified according to the *Cardiovascular and Interventional Radiological Society of Europe* (CIRSE) guidelines [10].

### Ancillary and forceps-assisted retrieval techniques

IVC filter retrieval utilizing forceps has been previously described, including case reports describing the technical process of filter engagement and extraction [6, 7, 11]. All procedures were performed under monitored anesthesia care or general anesthesia by an anesthesiologist. All removals were performed by an interventional radiologist. Sheath sizes and configurations, single or telescoping sheaths, as well as ancillary removal techniques were variable and based on operator preference [12, 13]. In all retrievals, after failure of ancillary techniques, including wire-loop snare engagement, hangman technique, balloon displacement, dual-venous access, and excimer laser dissection (Spectranetics CVX-300 Excimer Laser, Colorado Springs, CO, USA) [5], the 3-mm rigid endobronchial forceps (4162, LYMOL Medical, Woburn, MA, USA) or 2.25-mm myocardial forceps (flexible myocardial biopsy forceps; Cook Medical, Bloomington, IN, USA) were placed through the sheath and filter tip dissection was performed, if necessary, to disengage tip embedded filters from the wall of the IVC. The apex of the filter was then grasped with the forceps and retracted into the sheath. Patients with iliocaval thrombosis underwent subsequent iliocaval reconstruction with standard techniques. Post-retrieval fluoroscopy and venography were performed to evaluate for fractured fragments, caval spasm, pseudoaneurysm, or thrombosis. Patients were subsequently evaluated in the interventional radiology clinic, generally one month after filter retrieval.

### Statistical analyses

Calculations of mean, range, standard deviation, and percentages were performed using spreadsheet software (Excel 2017, Microsoft, Redmond, WA, USA).

## Results

### Type of “permanent” filters retrieved

**Fig. 2:**
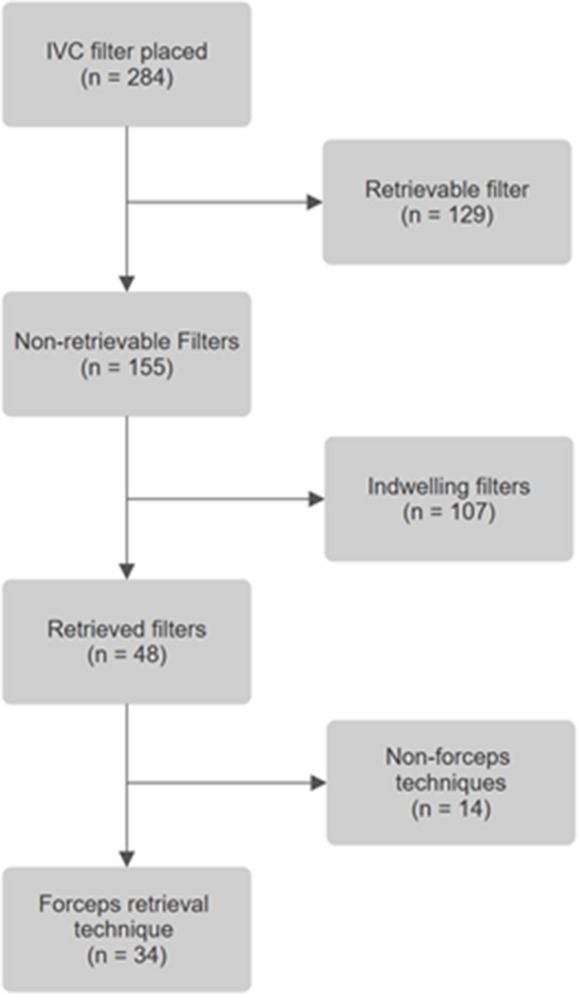
The distribution of retrieved “permanent” IVC filters with representative images of the deployed geometry. These filters lack a hook design found in retrievable filters. The strut vertex of these filters is targeted to facilitate collapse and removal

“Permanent” filters are shown in Fig. 2. Retrieved filters included Greenfield (*n* = 16; 47.1%), TrapEase (*n* = 10; 29.4%), Simon Nitinol (*n* = 5; 14.7%), VenaTech LP (*n* = 2; 5.9%), and Bird’s Nest (*n* = 1; 2.9%). Retrieval of the Bird’s Nest filter has been previously described [14]. The filters retrieved for strut fracture or migration included Greenfield (*n* = 2), Simon Nitinol (*n* = 2), VenaTech LP (*n* = 1), and Bird’s Nest (*n* = 1). A Greenfield filter was successfully retrieved prior to a planned open thoracoabdominal aortic aneurysm repair requiring venous cannulation for cardiopulmonary bypass.

### "Permanent" IVC filter retrieval techniques

**Fig. 3:**
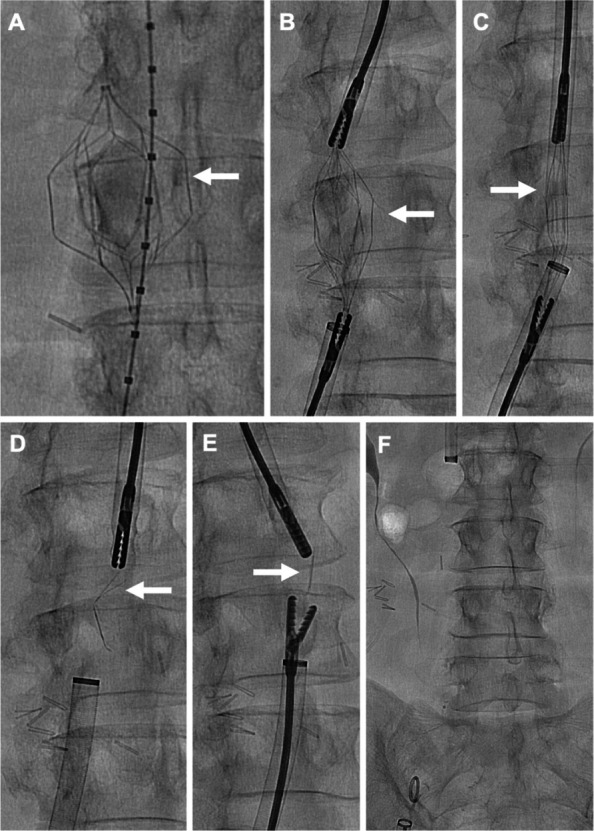
A 52-year-old female presented with a TrapEase “permanent” inferior vena cava filter and requested removal due to chronic back pain. **A** Fluoroscopic image showing the intact TrapEase inferior vena cava filter (white arrow). An Omni flush catheter is partially visualized. **B** 20-French sheaths were placed via right internal jugular vein and right common femoral vein accesses. Two pairs of endobronchial forceps were used to elongate and free the TrapEase inferior vena cava filter (white arrow). **C** The TrapEase inferior vena cava filter was further elongated (white arrow) and pulled into the right common femoral vein sheath. **D**, **E** During the retrieval process, two strut fractures (white arrows) were subsequently removed using the endobronchial forceps. **F** Fluoroscopic image showing all components of the TrapEase successfully removed

Endobronchial forceps retrieval is shown in Fig. 3. The endobronchial forceps were used in 30 (88.2%) patients, and the myocardial forceps were used in 4 (11.8%) patients. The median external sheath size placed was 16-French (range 10–22-French). Failed ancillary filter retrieval techniques utilized prior to forceps use included loop snare (*n* = 12; 35.3%), balloon-displacement (*n* = 5; 14.7%), dual-venous access (*n* = 4; 11.8%), laser dissection (*n* = 2; 5.9%), and hangman techniques (*n* = 1; 2.9%). More than one ancillary technique may have been employed during the same procedure. Thirteen (38.2%) retrievals did not attempt an additional ancillary technique.

“Permanent” IVC filter retrievals using endobronchial or myocardial forceps were technically successful in 88.2% of patients (*n* = 30). In one (2.9%) patient, the forceps were unable to safely dislodge a TrapEase filter and the sheath could not be advanced over the filter despite attempting to remove the filter from both the right internal jugular vein and the left femoral vein. Venography demonstrated a contained leak within the IVC at the level of the filter and, as a result, a 24-mm × 70-mm Wallstent endoprosthesis (Boston Scientific) was deployed across the indwelling IVC filter. Additional venography demonstrated an ongoing leak and a 21-mm × 10-mm stent-graft (TAG, W. L. Gore & Associates, Flagstaff, AZ, USA) was deployed within the indwelling Wallstent endoprosthesis across the excluded IVC filter. In the remaining 3 (8.8%) unsuccessful retrievals of a Greenfield, TrapEase, and Simon Nitinol filter, acute-on-chronic thrombus was identified during the procedure and the retrieval was aborted owing to risk for thromboembolism. All 3 patients were seen in the Interventional Radiology clinic and have been scheduled for venography with plans for repeat filter retrieval or iliocaval stent reconstruction with stent-assisted filter sequestration.

Mean fluoroscopy time was 75.1 min (range 10–230 min). Mean blood loss was 65 mL (range 5–300 mL).

### Immediate and long-term adverse events

A total of 30 (88.2%) patients had no major adverse events during the study period. Three (8.9%) patients developed IVC intimal tears resulting in contained leaks during retrieval of a Greenfield, TrapEase, and Bird’s Nest filter. Two of these patients were treated with stent placement and the third was treated with balloon inflation tamponade alone. The first was described above, the second occurred during the retrieval of a Greenfield filter and was repaired using an 18-mm × 60-mm Wallstent with thrombin injection between the interstices of the stent. There were no long-term complications related to these interventions. These were minor complications, CIRSE grade 1 [10]. One (2.9%) patient suffered a thromboembolic stroke following successful retrieval of a TrapEase filter which required mechanical thrombectomy. The patient’s hospital course was complicated by subarachnoid hemorrhage and pneumonia ultimately resulting in death 27 days after filter retrieval. This was a major complication resulting in patient death, CIRSE grade 6 [10].

### Mortality and follow-up evaluation

During the study period, there was one (2.9%) patient death. The mean follow-up duration was 647 days (range 27–2265 days).

## Discussion

The retrospective analysis demonstrated an 88.2% technical success rate in retrieving long-dwelling “permanent” IVC filters using the endobronchial or myocardial forceps with an associated adverse event rate of 11.8%. Since the first IVC filter was developed in 1967, the incidence of IVC filter placement has increased from an estimated 2000 placements in 1979 to 44,378 in 2015 among Medicare recipients [3, 15]. A large proportion of this increase came following the United States Federal Drug Administration (FDA) approval of the use of retrievable IVC filters in 2003 [16]. Because the original filter design was designed to be removed, the prevalence of “permanent” IVC filters remains unknown in the current population. Further research demonstrated the associated risks of long-dwelling “permanent” IVC filters, highlighting the need for filter retrieval when clinically indicated. There is a subset of patients who develop life-limiting comorbidities related to “permanent” IVC filters, when effective and safe filter retrieval techniques are clinically indicated.

One of the most frequently encountered IVC filter-related complications is iliocaval thrombosis. The observed rate of thrombotic occlusion of the IVC in patients with “permanent” filters is 6–30%; similar to the reported rate of 2–30% seen with retrievable filters [4, 17, 18]. Iliocaval thrombosis was the most frequently reported clinical indication for “permanent” filter retrieval in the current analysis, consisting of 67.6% of patients. The slightly higher rate of caval thrombosis may be related to the geometry of the filter as cylindrical (VenaTech LP and TrapEase) and umbrella element (Simon Nitinol) filters have been associated with higher rates of thrombosis than conical filters [4, 19, 20].

Another common complication of long-dwelling non-retrievable filters is strut fracture. The rate of filter fracture within the *Manufacturer and User Facility Device Experience* (MAUDE) database found a higher association with retrievable filters (95.4%) compared to “permanent” filters (4.6%) [21, 22]. Strut fracture is most commonly observed as a late complication after prolonged structural stress. The differences in strut fractures in retrievable and "permanent” filters are unknown, but may be related to design, material composition, or differences in surveillance. In this study, filter fracture and migration represented the second most common (17.6%) indication for “permanent” filter retrieval. When the clinical indication for removal is strut fracture, endobronchial and myocardial forceps are uniquely able to dissect and grasp chronically embedded filter struts, making them increasingly effective in filter retrieval cases with pre-existing strut fractures [23]. Zhong et al. has demonstrated in 535 patients that endobronchial forceps can be utilized to great effect for complex removal of temporary IVC filters with 98.7% technical success, and major side effects occurring in only 2% of patients [24]. Of the retrieved filters, 26% involved the removal of temporary filters with strut fractures.

In response to the 2010 FDA advisory statements recommending filter removal once the indication for protection has passed, the rate of filter removal procedures has increased from 7% in 2012 to 14% in 2015 [3, 25]. While many of the efforts to improve filter retrieval rates have focused on temporary filters, demonstrating safe and effective techniques to remove "permanent" filters remains important. Of the analyzed patients, 88% of the "permanent" filters were successfully removed using the endobronchial or myocardial forceps, similar to *Tamrazi *et al. in which retrieval of “permanent” filters was 91.6% (11/12) successful through the combination of endobronchial forceps (*n* = 6), wire-loop (*n* = 12), and laser sheath extractions (*n* = 8) [26]. Notably, the rate of technical success for retrieval in the current study was similar to the technical success rate of temporary filter retrieval. In a review by *Ni *et al. of 19 studies analyzing the efficacy of temporary filter retrieval, filters were successfully removed in 80–100% of cases [17]. The usefulness of the endobronchial forceps was further demonstrated by *Tavri* and colleagues in which 58/60 (96.7%) patients with a combination of "permanent" or temporary filters underwent successful filter retrieval with the rigid endobronchial forceps [6]. Further, successful filter retrieval with the forceps appears to be associated with experience, suggesting the presence of a learning curve for the technique [12].

Importantly, the reported 11.8% complication rate described in the present analysis is lower than that previously described of 16.7% in a sample of 12 “permanent” filter retrievals [26]. While previous analyses of filter retrievals utilizing the endobronchial forceps reported complication rates between 3.5 and 6.7%, only one "permanent" filter retrieval was attempted within these cohorts [6, 7]. Of note, longer-dwelling times, increased tilt angles, and hook embedment have been shown to significantly increase complication rates associated with filter retrievals, regardless of filter type, emphasizing the need for pre-retrieval imaging evaluation [27]. The 3 IVC intimal tears which occurred were likely attributable to the forceps dissection itself, but were treated immediately, without subsequent sequelae. The cerebral thromboembolism which occurred was presumed to be related to the filter retrieval given the temporality of the two events. However, a thorough cardiologic and neurologic evaluation failed to identify a clear etiology such as patent foramen ovale or other right-to-left shunt.

A previous report has described utilizing the myocardial forceps for the removal of a fractured strut; however, no previous reports have described the use of these forceps for retrieval of an entire filter [25]. The 4 filters removed with the myocardial forceps within this subgroup were all successfully retrieved without immediate complications. The smaller diameter of the myocardial forceps allows use of a smaller working sheath (7-French) as opposed to the 16-French sheath required for the endobronchial forceps.

There are several important limitations to the current study. First, this was a retrospective study performed over an 18-year period. The impact of evolving advanced techniques in aiding in the successful retrieval of “permanent” filters could not be independently assessed. The choice to use forceps, endobronchial or myocardial, during a retrieval was operator-dependent and may have been subjected to selection bias. Finally, imaging and clinical follow-up varied between patients, limiting the ability to identify all potential long-term complications.

Importantly, it should not be assumed that all “permanent” IVC filters should be retrieved when they are detected. Instead, a thorough, unbiased risk–benefit discussion should be held with the patient regarding the indications for filter retrieval, potential complications associated with retrieval, and potential filter-related complications associated with continued presence of the filter. The expanded technology available for filter retrieval, including endobronchial and myocardial forceps-assisted retrievals, has increased the feasibility of safely removing long-dwelling “permanent” filters in patients with iliocaval thrombosis, filter strut fracture, or when the patient desires removal. Future research into the clinical outcomes of patients with permanent IVC filter removal including symptom resolution and anticoagulation dosing.

## Conclusion

In the appropriate clinical scenarios, including iliocaval thrombosis, filter fracture, and patient request for retrieval, with benefits and risks discussed, a variety of long-dwelling “permanent” IVC filters may be removed using the 3-mm endobronchial and 2.25-mm myocardial forceps with a high rate of technical success.

## Data Availability

Raw data can be made available upon request.

## Abbreviations

IVC: Inferior vena cava
STROBE: Strengthening the Reporting of Observational Studies in Epidemiology
FDA: United States Federal Drug Administration
MAUDE: Manufacturer and User Facility Device Experience

## References

[1] DeYoung E, Minocha J. Inferior vena cava filters: guidelines, best practice, and expanding indications. Semin Intervent Radiol. 2016;33(2):65–70. 27247472 10.1055/s-0036-1581088 PMC4862857

[2] Duszak R, Parker L, Levin DC, Rao VM. Placement and removal of inferior vena cava filters: national trends in the medicare population. J Am Coll Radiol. 2011;8(7):483–9. 21723485 10.1016/j.jacr.2010.12.021

[3] Guez D, Hansberry DR, Eschelman DJ, Gonsalves CF, Parker L, Rao VM, et al. Inferior vena cava filter placement and retrieval rates among radiologists and nonradiologists. J Vasc Interv Radiol. 2018;29(4):482–5. 29305114 10.1016/j.jvir.2017.11.008

[4] Grewal S, Chamarthy MR, Kalva SP. Complications of inferior vena cava filters. Cardiovasc Diagn Ther. 2016;6(6):632–41. 28123983 10.21037/cdt.2016.09.08 PMC5220210

[5] Kuyumcu G, Walker TG. Inferior vena cava filter retrievals, standard and novel techniques. Cardiovasc Diagn Ther. 2016;6(6):642–50. 28123984 10.21037/cdt.2016.09.07 PMC5220200

[6] Tavri S, Patel IJ, Kavali P, Irani Z, Ganguli S, Walker TG. Endobronchial forceps-assisted complex retrieval of inferior vena cava filters. J Vasc Surg Venous Lymphat Disord. 2019;7(3):413–9. 30477980 10.1016/j.jvsv.2018.08.005

[7] Stavropoulos SW, Ge BH, Mondschein JI, Shlansky-Goldberg RD, Sudheendra D, Trerotola SO. Retrieval of Tip-embedded Inferior Vena Cava Filters by Using the Endobronchial Forceps Technique: Experience at a Single Institution. Radiology. 2015;275(3):900–7. 25581368 10.1148/radiol.14141420

[8] von Elm E, Altman DG, Egger M, Pocock SJ, Gøtzsche PC, Vandenbroucke JP. The Strengthening the Reporting of Observational Studies in Epidemiology (STROBE) statement: guidelines for reporting observational studies. Int J Surg. 2014;12(12):1495–9. 25046131 10.1016/j.ijsu.2014.07.013

[9] Hanauer DA, Mei Q, Law J, Khanna R, Zheng K. Supporting information retrieval from electronic health records: a report of University of Michigan’s nine-year experience in developing and using the electronic medical record search engine (EMERSE). J Biomed Inform. 2015;55:290–300. 25979153 10.1016/j.jbi.2015.05.003 PMC4527540

[10] Filippiadis DK, Binkert C, Pellerin O, Hoffmann RT, Krajina A, Pereira PL. Cirse Quality Assurance Document and Standards for Classification of Complications: The Cirse Classification System. Cardiovasc Intervent Radiol. 2017;40(8):1141–6. 28584945 10.1007/s00270-017-1703-4

[11] Workman C, Lewandowski R, Desai K. Techniques for retrieval of permanent inferior vena cava filters. Semin Interv Radiol. 2017;34(02):208–12. 10.1055/s-0037-1602597 PMC5467012 28607530

[12] Chick JFB, Stavropoulos SW, Shin BJ, Shlansky-Goldberg RD, Mondschein JI, Sudheendra D, et al. A 16-F sheath with endobronchial forceps improves reported retrieval success of long-dwelling “closed cell” inferior vena cava filter designs. J Vasc Interv Radiol. 2016;27(7):1027–33. 27241396 10.1016/j.jvir.2016.03.047

[13] Chen JX, Montgomery J, McLennan G, Stavropoulos SW. Endobronchial forceps-assisted and excimer laser-assisted inferior vena cava filter removal: the data, where we are, and how it is done. Tech Vasc Interv Radiol. 2018;2(2):85–91 (Epub 2018 Mar 9). 10.1053/j.tvir.2018.03.004 29784126

[14] Cooper KJ, Chick JFB, Srinivasa RN, Luhar A, Liles A, Moriarty JM. Retrieval of symptomatic Gianturco-Roehm Bird’s Nest inferior vena cava filters using rigid endobronchial forceps. Diagn Interv Imaging. 2018;99(12):829–30. 30539726 10.1016/j.diii.2018.07.006

[15] Stein PD, Matta F, Hull RD. Increasing use of vena cava filters for prevention of pulmonary embolism. Am J Med. 2011;124(7):655–61. 21592452 10.1016/j.amjmed.2011.02.021

[16] Franz RW, Johnson JD, Shah KJ. Symptomatic inferior vena cava perforation by a retrievable filter: Report of two cases and a literature review. Int J Angiol. 2009;18(4):203–6. 22477554 10.1055/s-0031-1278355 PMC2903035

[17] Ni H, Win LL. Retrievable inferior vena cava filters for venous thromboembolism. ISRN Radiol. 2013;22:2013. 10.5402/2013/959452 PMC4045516 24967292

[18] Deso SE, Idakoji IA, Kuo WT. Evidence-based evaluation of inferior vena cava filter complications based on filter type. Semin Intervent Radiol. 2016;33(2):93–100. 27247477 10.1055/s-0036-1583208 PMC4862854

[19] Corriere MA, Sauve KJ, Ayerdi J, et al. Vena cava filters and inferior vena cava thrombosis. J Vasc Surg. 2007;45(4):789–94. 17398389 10.1016/j.jvs.2006.12.048

[20] Simon M, Athanasoulis CA, Kim D, et al. Simon nitinol inferior vena cava filter: initial clinical experience. Radiology. 1989;172(1):99–103. 2662259 10.1148/radiology.172.1.2662259

[21] Andreoli JM, Lewandowski RJ, Vogelzang RL, Ryu RK. Comparison of complication rates associated with permanent and retrievable inferior vena cava filters: a review of the MAUDE database. J Vasc Interv Radiol. 2014;25(8):1181–5. 24928649 10.1016/j.jvir.2014.04.016

[22] Desai TR, Morcos OC, Lind BB, Schindler N, Caprini JA, Hahn D, et al. Complications of indwelling retrievable versus permanent inferior vena cava filters. J Vasc Surg Venous Lymphat Disord. 2014;2(2):166–73. 26993182 10.1016/j.jvsv.2013.10.050

[23] Dinglasan LAV, Trerotola SO, Shlansky-Goldberg RD, Mondschein J, Stavropoulos SW. Removal of fractured inferior vena cava filters: feasibility and outcomes. J Vasc Interv Radiol. 2012;23(2):181–7. 22178038 10.1016/j.jvir.2011.10.023

[24] Zhong A, Trerotola SO, Stavropoulos SW. Endobronchial forceps retrieval of embedded inferior vena cava filters: retrieval of 535 filters at a single center. J Vasc Interven Radiol. 2022;34(4):529–33. 10.1016/j.jvir.2022.12.005 36509239

[25] Food and Drug Administration. Removing retrievable inferior vena cava filters: initial communication. 2010. Available at: https://wayback.archive-it.org/7993/20161022180008/http:/www.fda.gov/MedicalDevices/Safety/AlertsandNotices/ucm221676.htm. Accessed 8 May 2019.

[26] Tamrazi A, Wadhwa V, Holly B, Bhagat N, Marx JK, Streiff M, et al. Percutaneous retrieval of permanent inferior vena cava filters. Cardiovasc Intervent Radiol. 2016;39(4):538–46. 26486152 10.1007/s00270-015-1214-0

[27] Al-Hakim R, Kee ST, Olinger K, Lee EW, Moriarty JM, McWilliams JP. Inferior vena cava filter retrieval: effectiveness and complications of routine and advanced techniques. J Vasc Interv Radiol. 2014;25(6):933–9. 24630748 10.1016/j.jvir.2014.01.019

